# Diversity and Disparity of Therocephalia: Macroevolutionary Patterns through Two Mass Extinctions

**DOI:** 10.1038/s41598-019-41628-w

**Published:** 2019-03-25

**Authors:** Henrik Richard Grunert, Neil Brocklehurst, Jörg Fröbisch

**Affiliations:** 1 Institut für Biologie, Humboldt-Universität zu Berlin, Invalidenstraße 42, Berlin, D-10115 Germany; 2 Museum für Naturkunde, Leibniz-Institut für Evolutions- und Biodiversitätsforschung, Invalidenstraße 43, D-10115 Berlin, Germany; 3 Department of Earth Sciences, University of Oxford, South Parks Road, Oxford, OX1 3AN UK

## Abstract

Mass extinctions have the potential to substantially alter the evolutionary trends in a clade. If new regions of ecospace are made available, the clade may radiate. If, on the other hand, the clade passes through an evolutionary “bottleneck” by substantially reducing its species richness, then subsequent radiations may be restricted in the disparity they attain. Here we compare the patterns of diversity and disparity in the Therocephalia, a diverse lineage of amniotes that survived two mass extinction events. We use time calibrated phylogeny and discrete character data to assess macroevolutionary patterns. The two are coupled through the early history of therocephalians, including a radiation following the late Guadalupian extinction. Diversity becomes decoupled from disparity across the end-Permian mass extinction. The number of species decreases throughout the Early Triassic and never recovers. However, while disparity briefly decreases across the extinction boundary, it recovers and remains high until the Middle Triassic.

## Introduction

Therocephalians were a diverse group of non-mammalian eutheriodont therapsids from the synapsid lineage of amniotes, first documented in the fossil record in the early Wordian^1^. They most likely originated in southern Gondwana, since early therocephalian fossils such as *Glanosuchus macrops* were found in the Beaufort Group of South Africa^1^ and later spread throughout the world^2^. The clade includes a great variety of insectivorous (e.g. Bauriamorpha), herbivorous (Bauriidae), and carnivorous species with greatly enlarged canine teeth in both jaws (e.g. Scylacosauridae)^3^. They are one of three therapsid lineages to survive the end-Permian mass extinction (EPME), the largest mass extinction in Earth’s history, thought to have wiped out up to 96% of all marine species and 70% of terrestrial vertebrate species^4–8^. The surviving vertebrates went through a series of diversifications afterwards to fill new vacant niches^6–9^.

The other two synapsid lineages which survived the EPME, the anomodonts and cynodonts, have been the subject of recent analyses examining their macroevolutionary patterns across this event^10,11^, with particular focus on the patterns of diversity (species richness) and disparity (morphological diversity) through time. In anomodonts, the most diverse and abundant clade of herbivores at the time, diversity and disparity were found to be decoupled, with substantial loss of diversity across the EPME, but a slow and continuous decline in disparity beginning earlier in the Permian^10^. In cynodonts, the lineage which eventually gave rise to mammals during the Triassic, conflicting results are found depending on the disparity measure employed: variance-based measures of disparity conflict with the diversity estimates, while range-based measures show a significant correlation^11^.

Many studies have found that species richness and disparity are often not correlated^12–14^. As a direct result of specialization and speciation, one could expect increasing disparity early in an evolutionary history, since clades tend to diversify along ecomorphological lines in a new environment^15^, leading to “early bursts” of morphological diversity independent of species richness^10,16,17^. It has been suggested that such decoupling can become more pronounced during periods of mass extinction, as if the extinction is non-selective or targets less specialised forms, disparity may remain high while diversity falls^17,18^.

We used discrete morphological characters to determine patterns of morphological variation within Therocephalia, allowing an examination of how it has changed throughout their history. By quantifying three different disparity metrics, we illustrate the pattern of therocephalian evolution and extinction across the Permian-Triassic boundary.

## Results

### Phylogenetic Relationships of Therocephalia

The analyses described below, both relating to diversity and disparity, incorporate a phylogenetic hypothesis. To account for uncertainty in the relationships of therocephalians, three sets of phylogenies were produced. All were based on the same character and taxon dataset^19^ and were inferred and time calibrated using the same fossilised birth death (FBD) model under the same parameters (see material and methods section), but different topology constraints were applied: (1) an entirely unconstrained analysis; (2) relationships constrained to those found in the parsimony analysis of Kammerer and Masyutin^19^ (although topology was free to vary within the polytomies found by that study) (3) relationships constrained to those found in the parsimony analysis of Liu and Abdala^20^ (again, topologies were free to vary within the polytomies identified by that analysis).

Although it is not the purpose of this study to examine phylogenetic relationships of Therocephalia in detail (and in any case the different topology constraints appear to have had minimal effect on the diversity and disparity estimates), there are some differences between the topologies of the maximum clade credibility trees resulting from these analyses that warrant discussion.

**Figure 1:**
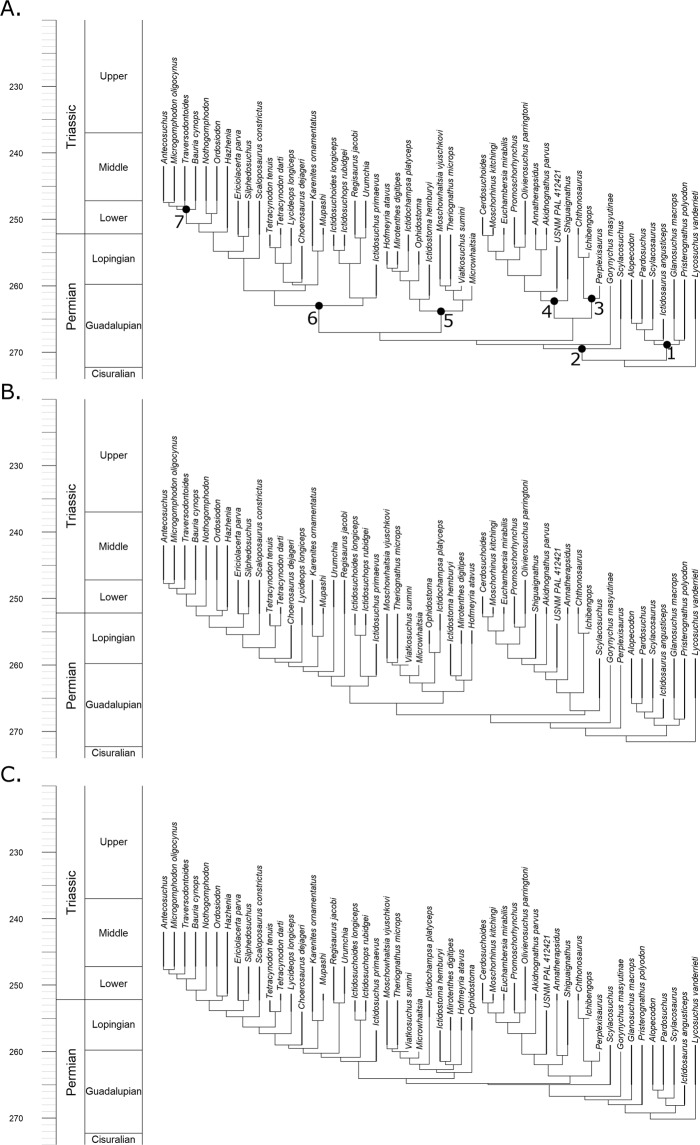
Maximum clade credibility trees of Therocephalia identified by the fossilised birth death skyline model. (**A**) Tree produced by analysis with no topology constraint. (**B**) Topology constrained to that found by Kammerer and Maysutin^19^. (**C**) Topology constrained to that found by Liu and Abdala^20^.

The parsimony analysis of Liu and Abdala^20^ was unable to resolve the relationships among the Scylacosauridae, an assemblage of basal taxa found early in the fossil record of therocephalians. The modifications made by Kammerer and Masyutin^19^ found a monophyletic Scylacosauridae containing six species under parsimony analysis. The maximum clade credibility tree from reanalysis of this data matrix here using the FBD model also finds a monophyletic Scylacosauridae when no topology constraints are applied (Fig. 1A). However, when the FBD model is applied with the topology constrained to those found by Liu and Abdala^20^, Syclacosauridae is found to be paraphyletic (Fig. 1C), with *Pristerognathus* and *Glanosuchus* found more closely related to Eutherocephalia than other scylacosaurids. This result is somewhat paradoxical: one might expect the analysis where no topology constraints are applied to correspond more with the topology where scylacosaurid relationships are less constrained (that of Liu and Abdala).

In other aspects, the early diverging nodes of the unconstrained analysis diverge from the topology constrained to that of Kammerer and Masyutin. The unconstrained analysis and that of Liu and Abdala found *Scylacosuchus* to lie outside the Eutherocephalia. The parsimony analysis of Kammerer and Masyutin^19^ was unable to resolve the early divergences of the Eutherocephalia but found *Scylacosuchus* within this clade (albeit in a polytomy with most of the whaitsioids). *Perplexisaurus*, however, was found by Kammerer and Masyutin to be outside Eutheroocephalia, but Liu and Abdala found it nested deep within this clade, a member of the family Chthonosauridae. The unconstrained FBD analysis again agrees with Liu and Abdala.

Relationships elsewhere in the three maximum clade credibility trees are largely consistent with each other, although there is some disagreement regarding the earliest divergences of the Bauriodea. Liu and Abdala^17^ were unable to resolve a monophyletic Ictidosuchidae (*Ictidosuchus*, *Ictidosuchoides* and *Ictidosuchops*) but did find a monophyletic Regisauridae (*Regisaurus* and *Urumchia*). Conversely, Kammerer and Masyutin^16^ found a monophyletic Ictidosuchidae but a paraphyletic Regisauridae. The unconstrained analysis here provides a novel set of relationships: Regisauridae is found within a paraphyletic Ictidosuchidae. This result is perhaps driven by the incorporation of tip ages into the FBD analysis: the relationships of Ictidosuchidae and Regisauridae in the unconstrained analysis are the most stratigraphically consistent, with the earlier appearing Ictidosuchidae forming successive outgroups to the Triassic Regisauridae.

### Diversity

**Figure 2:**
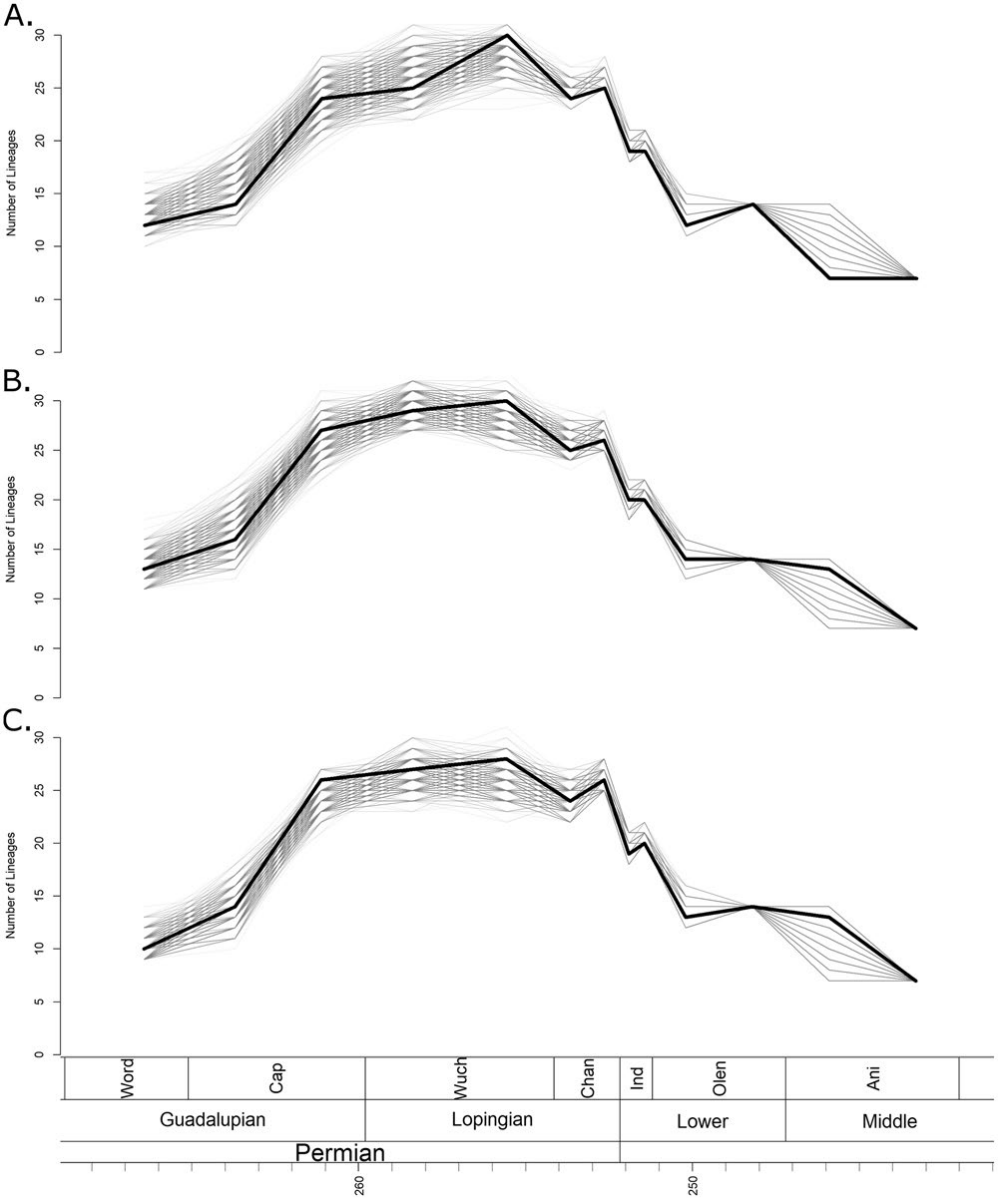
Phylogenetic diversity estimates of Therocephalia. The thick black line represents the diversity estimated using the maximum clade credibility tree, and the thin grey translucent lines indicate diversity estimated from 1000 trees drawn at random from the posterior distribution. (**A**) Diversity estimate based on trees produced by analyses with no topology constraints. (**B**) Based on trees constrained to topology found by Kammerer and Maysutin^19^. (**C**) Based on trees constrained to topology found by Liu and Abdala^20^.

Phylogenetic diversity estimates calculated using the maximum clade credibility (MCC) trees identified by the three FBD analyses (Fig. 1) all indicate that diversity remained low until the late Capitanian (the latest stage of the Guadalupian) (Fig. 2). Diversity then increased rapidly, before remaining relatively constant throughout the Lopingian. When the tree produced by the unconstrained tip dating analysis is used, a slight further increase in diversity during the late Wuchiapingian is indicated (Fig. 2A), although this peak is absent in the trees produced by the topologically constrained analyses (Fig. 2B,C). This inconsistency is likely due to the fact that both the MCC trees produced in the constrained analyses support an earlier radiation of the Baurioidea during the late Guadalupian, while the unconstrained MCC indicates a later diversification of this group.

1000 randomly selected trees from the three posterior distributions produced by the tip dating analyses (Supplementary Data S1) also support an increase between the Guadalupian and the Lopingian, although there is variation in the pattern identified; whether the increase was stepwise or gradual is unclear (Fig. 2). However, a substantial increase between the early and late Capitanian is universally identified. The large range of diversity estimates obtained from the different posterior distributions during the late Guadalupian and early Lopingian may be due to the uncertainty surrounding the relationships of Scylacosauridae and early Eutherocephalia already discussed; it should be noted that the ranges are lower in the results from the trees with constrained topologies (Fig. 2B,C).

At the Permian-Triassic boundary, the phylogenetic diversity estimates calculated from all 1001 chosen phylogenies from all three tip dating analyses become considerably less variable (Fig. 2). The universal pattern identified is a decrease in species richness beginning across the Permian-Triassic boundary and continuing until the early Olenekian. While diversity does recover slightly in the late Olenekian, the Permian richness is never again reached. During the Anisian, diversity again declines, and therocephalians disappear from the fossil record in the Ladinian.

### Disparity

**Figure 3:**
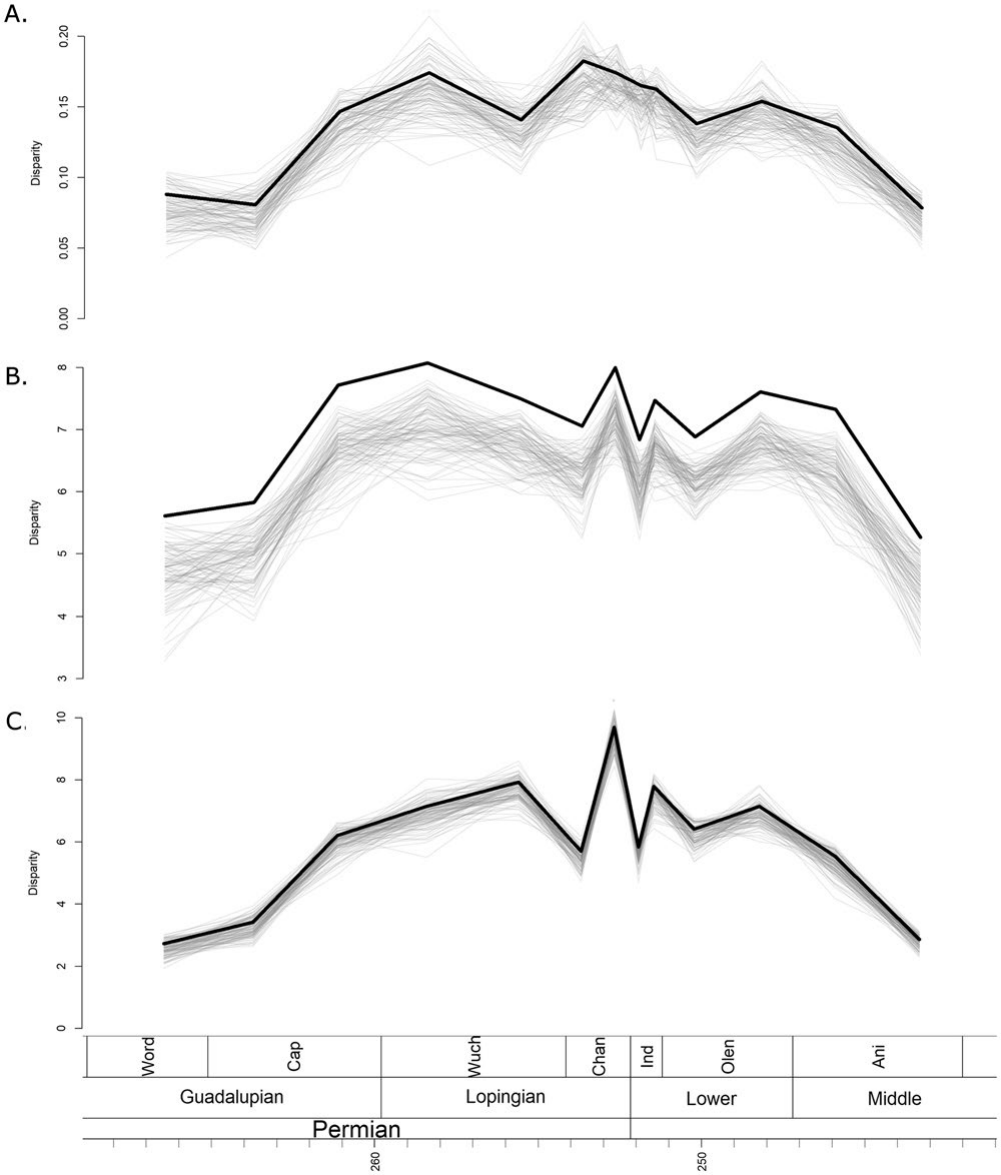
Disparity through time estimated from the maximum clade credibility tree of the unconstrained analysis. The thick black line represents the raw disparity estimate, the thin translucent grey lines represent disparity estimates from 100 taxonomic bootstrapping replicates. (**A**) Disparity measured using the sum of variance; (**B**) Disparity measured using sum of ranges; (**C**) Disparity measured using mean distance from the centroid.

Disparity was calculated using three metrics: the sum of variances (SOV), sum of ranges (SOR) and mean distance from centroid (DFC). The disparity curves calculated incorporated information on the ancestral morphologies deduced from the phylogenies (see Materials and Methods for more detail), and so disparity curves were calculated using all three phylogenetic hypotheses. However, the differences in the three sets of disparity curves were minimal (Figs 3 and S1, S2), so for the rest of the paper the disparity curves under discussion are those inferred from the tree produced by the unconstrained FBD analysis

All three disparity metrics, the sum of variances (SOV), sum of ranges (SOR) and mean distance from centroid (DFC) indicate a low initial disparity, which rises rapidly in the late Capitanian and then plateaus for much of the Lopingian (Fig. 3). When taxon bootstrapping is applied, this pattern is consistently observed when using the SOR and DFC measures of disparity (Fig. 3B,C), but the SOV measure shows much greater variation in the patterns identified (Fig. 3A).

Across the EPME, the raw SOV remains relatively stable, with only a slight decrease persisting into the Triassic (Fig. 3A). Again, bootstrapping the data leads to considerably more uncertainty, with a variety of trends observed and a wide range of disparity estimates. The SOR and DFC measures show similar changes across the EPME, which are robust to bootstrapping (Fig. 3B,C). Immediately prior to the extinction, in the late Changhsingian, both curves show a peak in disparity. Across the Permian-Triassic boundary both exhibit a significant decrease, but in the late Induan both rise again. During the Triassic, the SOR and DFC measures show a slight rise to a plateau in the Olenekian, but both decrease in the late Anisian prior to the extinction of Therocephalia.

### Statistical tests

The strength of the correlations between the diversity and disparity varies depending on the metric applied. The correlation when SOV is used to measure the disparity is weak, but significant correlations are observed when SOR and DFC are used (Table 1).

**Table 1:** Results of statistical comparisons.

Comparison	Statistical test	Test statistic	P value
Diversity~Disparity (SOV)	Spearman’s Rank	0.1328671	0.6834
Diversity~Disparity (SOR)	Spearman’s Rank	0.5944056	0.04575
Diversity~Disparity (DFC)	Spearman’s Rank	0.7762238	0.00466
Bootstrapped disparity before EPME~after EPME (SOV)	Wilcoxan Signed rank test	2415	0.0616720
Bootstrapped disparity before EPME~after EPME (SOR)	Wilcoxan Signed rank test	9960	1.017800ex 10^−32^
Bootstrapped disparity before EPME~after EPME (DFC)	Wilcoxan Signed rank test	10000	3.074572 × 10^−33^

When the bootstrapped disparities before the EPME are compared to those after, the significance of the change also varies depending on the disparity metric. The SOV indicates no significant change (possibly due to the large variation in patterns observed from the bootstrap data), but highly significant decreases are identified when the SOR and DFC are used.

## Discussion

### Comparison with Previous Analyses

Huttenlocker and Smith^21^ carried out a preliminary assessment of therocephalian diversity through time, as well as estimating origination and extinction rates through time. They used the results of this study to support a gradual rise of therocephalian diversity throughout the Permian. Origination and extinction rates were also inferred to rise continuously throughout the Permian, indicating a pattern of constant turnover with new species constantly and rapidly replacing the old.

The diversity curve of Huttenlocker and Smith^21^ contradicts the curve presented here particularly regarding the early evolution of therocephalians. We find, rather than a gradual increase in species richness throughout the Permian, instead a rapid increase in the latest Guadalupian is supported by the maximum clade credibility trees. The more rapid radiation in the late Guadalupian depicted in our study is due to the extension of multiple origination events back into the Capitanian stage. Huttenlocker and Smith^21^ argued for a “long fuse” model of therocephalian evolution, where the origin of clades such as the whaitsioids and the baurioids occurred in the Guadalupian, but the clades did not diversify until later. The tip dating analysis, on the other hand, suggests a “shorter fuse”, at least for the whaitsioids. The bulk of their diversification appears to have occurred by the end of the Guadalupian (Fig. 1), contributing to the more rapid rise in diversity in the late Capitanian. In fact, the bauriods may also have a shorter fuse as well; while the unconstrained FBD tree suggests a Lopingian diversification of this clade (Fig. 1A), when the topologies are constrained to those found by previous studies much of the diversification of bauriods occurred in the late Guadalupian (Fig, 1B,C), over a similar timescale to that of the whaitsoids.

The difference may also reflect the different methods employed to assess diversity. While Huttenlocker and Smith^21^ do not describe the method used to infer their diversity estimate, comparing the richness values exhibited in their Figure 11 to the lists of taxa and their ranges presented in their Tables 2 and 3, it appears that the diversity estimate represents a taxic diversity estimate; that is, a raw count of species. Such an estimate would be heavily influenced by the substantial sampling biases prevalent in the Permian-Triassic terrestrial record^22–25^. The phylogenetic diversity estimate employed here endeavours to include unsampled portions of the fossil record (inferred from the phylogeny) in the diversity estimate, and has been shown by multiple simulation studies to more closely represent the true diversity than the taxic diversity estimate^26,27^. Moreover, a more robust tip dating approach to generating the phylogenies, incorporating estimates of rates of origination, extinction, sampling and character change (see materials and methods) provides a more reliable indication on the origination age of clades than the observed fossil record.

### Radiation Following the End-Guadalupian Extinction

Adaptive zone invasion is hypothesised to coincide with increased rates of both speciation and morphological evolution. Radiation into novel or under-exploited adaptive landscapes should maximise selection for divergence^28,29^. It is hypothesised that, as the lineage diversified rapidly along ecological lines, morphological diversity should accumulate rapidly early in the clade’s history: an “early burst” model. An examination of palaeontological data^15^, suggested that early increases in disparity are the most frequently observed pattern.

Previous examinations of synapsid diversity and disparity have supported an early burst model. Anomodont evolution was characterised by an initial peak in disparity followed by a slow decline^10^. Cynodonts also exhibit their most rapid rates of morphological diversification early in their history^11^. On the other hand, eureptile clades appear to have experienced delayed radiations. Archosauromorphs were found to have low disparity and rates of evolution early in their history, but radiated in the aftermath of the EPME, presumably filling the newly vacated areas of ecospace^8^. Captorhinidae also show this pattern: the peak in disparity and rates of evolution occur during the Kungurian and Roadian, despite the clade originating in the Carboniferous^30,31^. This was attributed to the late exploration of novel regions of ecospace – the herbivorous and omnivorous diet. Therocephalians also show relatively late exploration of novel diets (herbivorous members do not appear until the Triassic), and so their macroevolutionary patterns might be expected to be more similar to that of Captorhinids than of the more closely related synapsids.

The early evolution of Therocephalia exhibits a slower increase in disparity than those observed in anomodonts and cynodonts. Although they first appear in the fossil record during the Wordian, the greatest increase in all three disparity metrics does not occur until the late Capitanian, the last substage of the Guadalupian. A plateau is reached during the early Wuchiapingian, although the SOV measure of disparity indicates a temporary trough during the late Wuchiapingian (Fig. 3A). The increase in disparity does not precede the increase in diversity as in anomodonts, but instead both increase over a similar time.

The delayed diversification of Therocephalia, both of morphology and species richness, may be linked to the middle Permian mass extinction. A late Guadalupian mass extinction has been recognised both in the marine and terrestrial realms^32–37^. Using data on the tetrapods of the Karoo, it was demonstrated that the extinction on land occurred at the end of the *Tapinocephalus* Assemblage Zone (AZ), which radiometric ages suggested was before the end of the Capitanian^38^. In fact, their radiometric dates suggest that the *Pristerognathus* AZ (the post-extinction assemblage) falls entirely within the Capitanian. The principle victims of the mass extinction were the the Dinocephalia^38^, including the carnivorous Anteosauridae, and the predominantly (although not exclusively) herbivorous Tapinocephalia.

**Figure 4:**
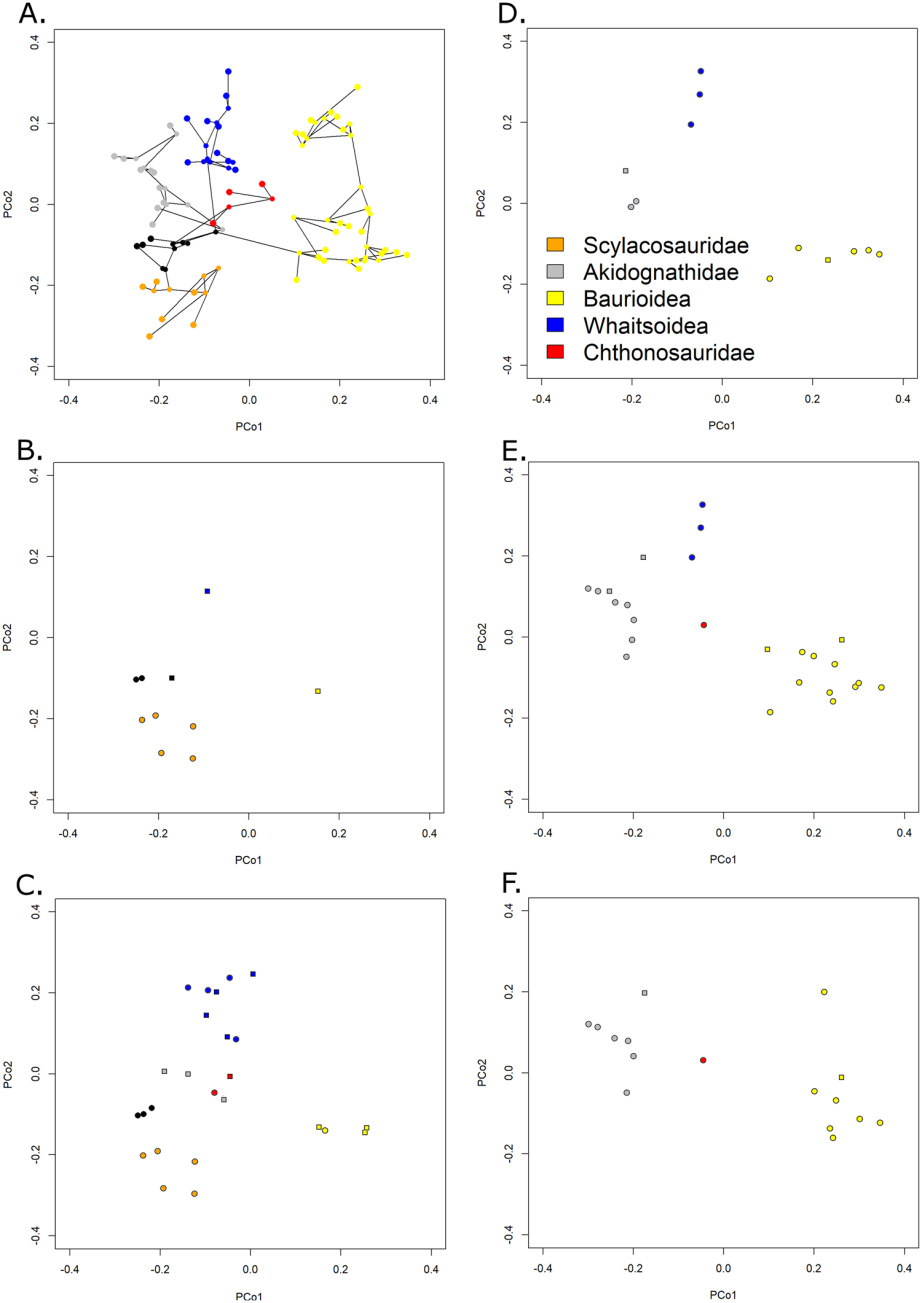
Morphospace occupation of Therocephalians through time. (**A**) Phylomorphospace of all therocephalians, showing the position of both tips and internal nodes along Principal coordinates 1 and 2. Phylogeny is the maximum clade credibility tree of the unconstrained analysis. Colours represent affinities (those in black are not assigned to one of the five clades indicated in the legend). (**B**) Morphospace indicating the position of tips and internal nodes of therocephalians from the early Capitanian. Filled circles represent tips, squares represent internal nodes. (**C**) Late Capitanian morphospace. (**D**) Early Changhsingian morphospace. (**E**) Late Changhsingian morphospace. (**F**) Early Induan morphospace.

The late Capitanian diversification of Therocephalia may represent an adaptive radiation following the extinction event. According to the maximum clade credibility tree produced by tip dating (Fig. 1), it is at this time that the major therocephalian clade, the Eutherocephalia, diversified. The Whaitsioidea appear and radiate during the latest Guadalupian. When one examines the changes in morphospace occupation between the early and late Capitanian (Fig. 4B,C), the increase in disparity is seen to be driven predominantly by the whaitsoids. The Akidnognathidae and Baurioidea also appear to have their roots in the late Capitanian according to the tip dating analysis, although their radiation does not occur until later. The rapid diversification of these clades, driving increases in both disparity and diversity, may represent an ecological release of large carnivorous therocephalians following the demise of the previous dominant predators, the Anteosauridae.

### Decoupling of Diversity and Disparity across the EPME

The impact of the end-Permian mass extinction on terrestrial vertebrates has been assessed in a large number of papers over the last decade^10,24,25,39–42^. These studies have highlighted several consistent patterns. First, the extinction was particularly severe for synapsids^24,41^, whose extinction rates were more than double those of both parareptiles and eureptiles^41^. Second, the recovery period of the extinction was unusually long, with continued ecosystem instability and elevated extinction rates throughout the Early Triassic^41–43^.

Among synapsids, the lineages terminated at the EPME were the two predatory lineages Biarmosuchia and Gorgonopsia. The cynodonts, of low diversity during the Permian, radiated both in terms of species richness and morphological diversity in the aftermath of the extinction^11,44^. The anomodonts were of extremely high species richness during the Permian, but their morphological diversity had been declining since the Guadalupian^10^. The decline in disparity does not seem to have been accelerated by the EPME, but species richness decreased dramatically and did not recover until the Middle Triassic.

The EPME represents the largest decrease in numbers of therocephalian species (Fig. 2). All 1001 tested phylogenetic diversity estimates support a species richness decline first across the Permian-Triassic boundary. The decline continues throughout the Early Triassic, with the number of lineages decreasing by more than half between the late Changhsingian and the early Olenekian. The Whaitsioidea, the most diverse lineage in the Lopingian, was entirely wiped out, and although the herbivorous Bauriidae radiated in the aftermath, therocephalian richness never returned to the levels observed in the Permian.

The SOR and DFC disparity curves of Therocephalia correlate significantly with the species richness curve (Table 1), and similarities may be observed before the latest Lopingian (rises in the late Capitanian to Wuchiapingian plateaus) and after the Induan (late Olenekian rises and late Anisian falls). However, diversity and disparity appear to have become decoupled in the stages surrounding the extinction events, and all three disparity curves contrast strongly with the species richness patterns. The SOV curve exhibits no significant change across the extinction event and remains high until the Middle Triassic (Fig. 3A). The SOV and DFC both increase immediately prior to the extinction in the late Changhsingian (Fig. 3B,C). There is a significant decline across the Permian-Triassic boundary (Table 1), but an almost immediate recovery during the Induan. By the Middle Triassic, both had returned to the levels of disparity observed prior to the extinction.

The contrasting patterns observed in the different disparity curves allow comment on the pattern of selectivity of the EPME. The increase in SOR and DFC immediately prior to the extinction indicates an increase in the total morphospace occupation, although the lack of change in the SOV and species richness suggests that this is due to a very small number of “specialists”; the bulk of the taxa continue to occupy the same region of morphospace. Across the EPME, there is again not substantial change in the SOV, but a significant SOR and DFC reduction. Two possible patterns are consistent with this observation: (1) the new specialists which appeared in the late Changhsingian were immediately culled by the extinction, leaving the original area of morphospace unchanged; (2) the original area was culled, and the new lineages diversified to occupy a volume of morphospace comparable to the earlier Lopingian values. The pattern of extinction observed in Therocephalia is more consistent with the latter explanation. The Whaitsioidea, which comprised the bulk of therocephalian diversity throughout the Lopingian, were eliminated in the EPME, while the Akidnognathidae and Baurioidea, which radiated in the latest Permian, survived into the Triassic (Fig. 1). This may be seen in the morphospace diagrams (although one should be careful of interpreting only two principal coordinate axes). Between the early and late Changhsingian, the baurioids and akidnognathids are the two clades that diversify, increasing morphospace occupation at both ends of PCo1 (Fig. 4D,E). During the Induan, both clades remain in similar regions of morphospace, but the whaitsoids disappear, considerably reducing disparity along PCo2 (Fig. 4F).

The Baurioidea make up the bulk of therocephalian diversity during the Triassic. Although their species richness never reaches that of the Permian, they include a wide range of morphologies (although they are restricted to small sizes), including herbivorous and potentially semi-aquatic forms^43,45^, and account for the morphological diversity of the Triassic being nearly equal to that of the Permian. However, by the middle of the Triassic they were very much in decline, restricted to the herbivorous Bauriidae, and all three disparity metrics show a decrease in the late Anisian (Fig. 3). Therocephalians are not known from beyond the Anisian, although their disappearance does not appear to be associated with any mass extinction event, but rather with an extended episode of elevated extinction rates and declining diversity that affected synapsids throughout the Middle and Late Triassic^10,41^.

## Conclusions

The results presented here suggest that taxonomic diversity and discrete character disparity were correlated during the initial adaptive radiation of therocephalians but became decoupled during the EPME. The Permian-Triassic mass extinction only had a dramatic impact on therocephalian species richness, but morphological diversity was largely unchanged and remained high for 10 million years after (although the number of species never fully recovered). A greater impact on their morphological evolution was caused by the end-Guadalupian extinction, which allowed an “ecological release” causing the taxa to diversify both in terms of number of species and morphological diversity.

## Materials and Methods

### Phylogeny and Character Data

The dataset used in this study was taken from the most recent and comprehensive phylogenetic analysis of therocephalians^19^. The data matrix includes 49 therocephalian taxa with 136 discrete characters representing morphological variations across the entire skeleton. The first appearance date (FAD) and last appearance date (LAD) in millions of years ago for all taxa within the phylogeny were compiled from the Paleobiology Database, downloaded via the Fossilworks platform (www.fossilworks.org). Ranges of species are treated as continuous from the first to last appearance, ignoring possible gaps in the fossil record. The species ranges are presented in Supplementary Table S1.

In order to produce a phylogenetic framework for the diversity analysis, a Bayesian tip-dating approach was employed that uses the character data and the ages of the tip taxa to simultaneously infer the phylogenetic relationships and node ages: the Fossilised Birth Death (FBD) model^46,47^. This method was implemented in MrBayes 3.2.6^48^. An offset exponential root prior was applied, with a minimum age 1 million years before the first appearance of therocephalians and a mean age at the Cisuralian/Guadalupian boundary. The choice of prior is based on previous analyses that have suggested a major turnover in dominant fauna at this time, along with the principal diversification of therapsids^39,41,49^ and so this is not an unreasonable time to expect therocephalians to have originated. In any case this age is not a hard lower bound; the exponential root prior allows root ages to be extended beyond the mean of the distribution. The analysis was run four times for 10,000,000 generations, sampling every 1000, thus producing a posterior distribution of 40,000 trees. 25% of these were discarded as burn-in. The full posterior distribution of trees before burnin is found in Supplementary Data S1, and the maximum clade credibility tree in Fig. 1A.

Due to disagreement and uncertainty regarding the relationships within therocephalians in recent studies, two further phylogenetic analyses were carried out. The parameters were identical to those described above, with the exception that the topology was constrained to represent the topologies found in two recent studies. The first topology constraint is that found by a parsimony analysis by Kammerer and Masyutin^19^, the second is that found by a parsimony analysis of an older version of the same matrix by Liu and Abdala^20^. Kammerer and Masyutin were unable to resolve the relationships of the early diverging eutherocephalian clades relative to each other, and indeed failed to resolve a monophyletic Whaitsioidea. Liu and Abdala^20^ were able to resolve these relationships, although they found Syclacosauridae to form a polytomy at the base of the therapsid tree, while Kammerer and Masyutin^19^ found this clade to be monophyletic. The maximum clade credibility trees of these analyses are shown in Fig. 1B,C, and the posterior distributions of trees are presented in Supplementary Data S2 and S3.

It should be noted that the matrix originally used by Liu and Abdala^20^ was not identical to that of Kammerer and Masyutin^19^; these authors made certain changes, including the removal of autapomorphies. Moreover, Liu and Abadala’s matrix did not include the later-described taxon *Gorynychus*. Because of the removal of autapomorphies, important both for tip-dating and disparity analyses^50,51^, it was decided not to examine disparity using Liu and Abdala’s matrix, but to instead, as described above, carry out the tip-dating and disparity analyses using the Kammerer and Masyutin^19^ matrix, but to test the impact of constraining the topology to that found by Liu and Abdala^20^.

### Diversity

Phylogenetic diversity estimates were calculated as species richness estimated using the phyloDiv function as implemented in R package paleotree^52^. This method accounts for the incomplete sampling of the fossil record by including ghost lineages (lineages not sampled in the fossil record but inferred to have been present from the phylogeny) in the diversity estimate^26,27^. To account for uncertainty in the ages of lineages and phylogenetic relationships, phylogenetic diversity estimates were calculated for the three maximum clade credibility trees, as well as a random selection of 1000 trees from each of the Bayesian posterior distributions.

The period of time under study was split into 12 time bins, from the first appearance of therocephalians in the fossil record in the Wordian until their extinction in the Anisian. The time bins represent substages: each international stage was split into two, with the boundary at the midpoint of the stage.

### Disparity

The 130 discrete morphological characters from the character matrix used to infer the phylogeny were also used to represent morphology in calculating disparity through time (note that as this matrix was designed for inferring phylogeny, the characters are only those considered parsimony informative). As well as the character scores provided for the tip taxa in the character matrix, character scores were obtained for the nodes within the tree with maximum-likelihood ancestral state reconstruction using functions in the R package Claddis^53^. The internal nodes could therefore be included as data points in the disparity calculation, thus allowing for ancestral morphologies which may have been outside the range of morphologies observed in the descendants^54^. We computed the pairwise morphological distances between each taxon as Gower dissimilarity, and performed a multi-dimensional scaling (principal coordinates analysis), again in Claddis. This produces a series of linearly uncorrelated continuous measurements representing each taxon’s position in morphospace.

The disparity through time was calculated using the same bins as diversity through time, using the R package dispRity^55^. This package allows the incorporation of information from the phylogeny into disparity studies, using the morphologies inferred from the internal nodes (described above).

The disparity of the principal coordinate values in each time bin was assessed using three metrics: sum of ranges (SOR), sum of variances (SOV) and mean Euclidean distance of each taxon from the centroid (DFC). SOR and DFC are measures of the total morphospace occupied but are vulnerable to sample size^12^ and morphologically distant singular taxa^56^ but this may be compensated by bootstrapping the data in dispRity. SOV is a measure of the spread of the data within the total morphospace. For each of the three disparity measures, 100 taxon bootstrapping replicates were carried out. The resilience of the methods used to sampling heterogeneity may be shown by correlation tests between the bootstrapped disparity curves and the observed sample size in each time bin using the spearman’s rank correlation coefficient. Only three of the 100 SOV curves showed significant (p < 0.05) correlation with sample size (Supplementary Data S4). The range-based metrics performed less well; 15 of the SOR curves and 13 of the DFC curves showed a significant correlation with sample size (Supplementary Data S5 and S6). Nevertheless, the overwhelming majority of all curves did not, indicating that bootstrapping and incorporation of information from the phylogeny go some way to alleviating the impact of sampling heterogeneity.

### Statistical Tests

The correlation between the three disparity curves and the diversity estimate were calculated using the Spearman’s rank correlation coefficient in R. Prior to the correlation test, the time series were corrected using generalised differencing^57^ to account for autocorrelation (the temporal non-independence of the data). To examine the significance of disparity changes across the Permian-Triassic boundary, disparity values of the 100 bootstrap replicates of the two time bins either side of the extinction event were compared using a Wilcoxan Signed Rank Test test.

## Supplementary information

Suppementary Figures and Tables

Supplementary Dataset 1

Supplementary Dataset 2

Supplementary Dataset 3

Supplementary Dataset 4

Supplementary Dataset 5

Supplementary Dataset 6

## Data Availability

All data is included in the Supplementary Data Files.
