# Supplementary material for: Diversity and Disparity of Therocephalia: Macroevolutionary Patterns through Two Mass Extinctions
Source: Sci Rep. 2019 Mar 25;9:5063. doi: 10.1038/s41598-019-41628-w (PMC6433905; doi:10.1038/s41598-019-41628-w)
Supplement: Supplementary file 1 — Suppementary Figures and Tables [file 41598_2019_41628_MOESM1_ESM.docx]

Electronic Supplement

Diversity and Disparity of Therocephalia: Macroevolutionary Patterns through Two Mass Extinctions

Henrik Richard Grunert^a,b^, Neil Brocklehurst^b,c^*, Jörg Fröbisch^a,b^

^a^ *Institut für Biologie, Humboldt-Universität zu Berlin, Invalidenstraße 42, Berlin D-10115, Germany*

^b^ *Museum für Naturkunde,* *Leibniz-Institut für Evolutions- und Biodiversitätsforschung, Invalidenstraße 43, D-10115 Berlin, Germany*

^c^ *Department of Earth Sciences, University of Oxford, South Parks Road, Oxford, UK, OX1 3AN*

* Corresponding Author: Neil Brocklehurst, neil.brockehurst@earth.ox.ac.uk

Supplementary Figure 1


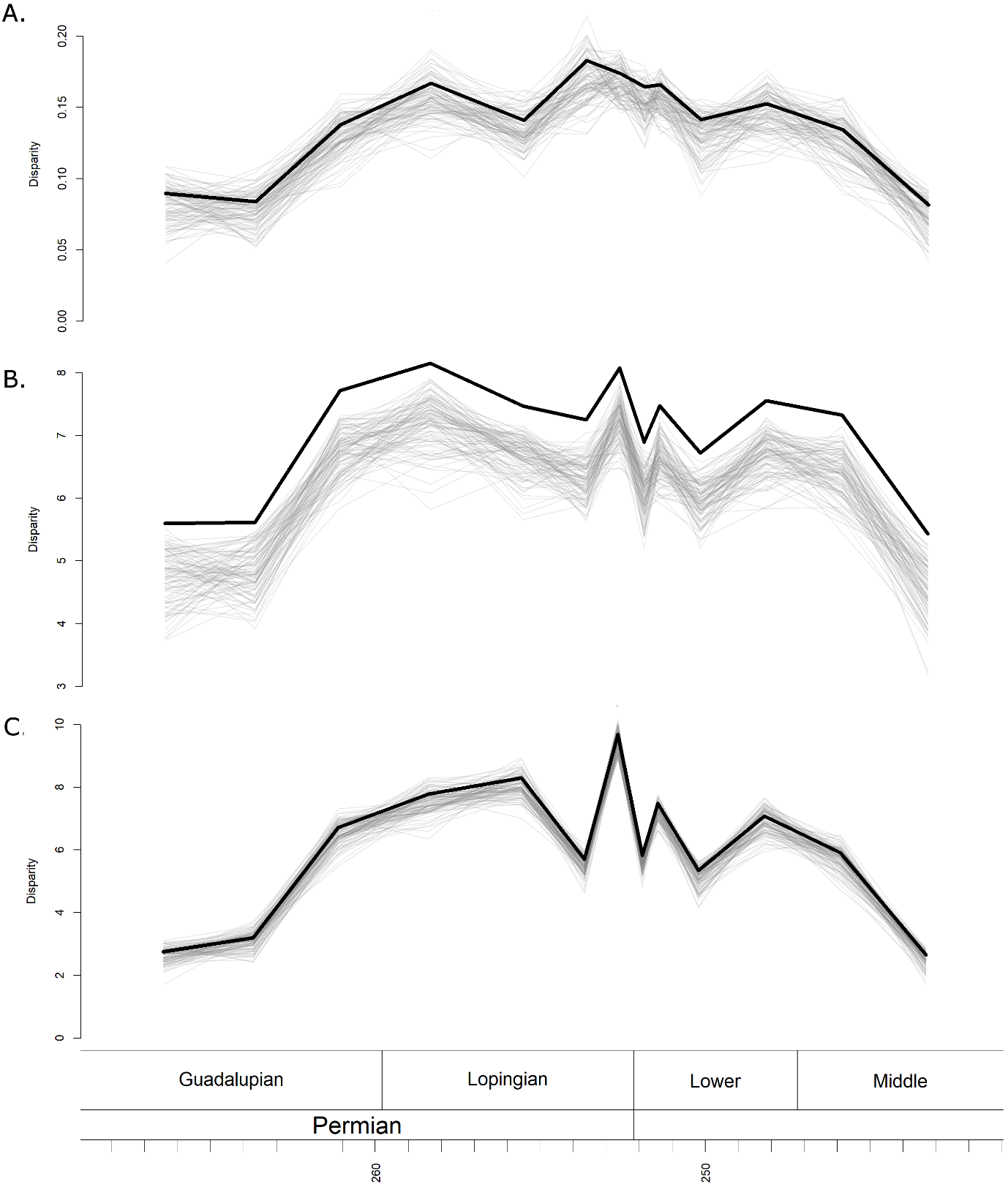


Disparity through time estimated from the maximum clade credibility tree of the analysis where topology is constrained to that of Kammerer and Masyutin [1]. The thick black line represents the raw disparity estimate, the thin translucent grey lines represent disparity estimates from 100 taxonomic bootstrapping replicates. A) Disparity measured using the sum of variance; B) Disparity measured using sum of ranges; C) Disparity measured using mean distance from the centroid.

Supplementary Figure 2


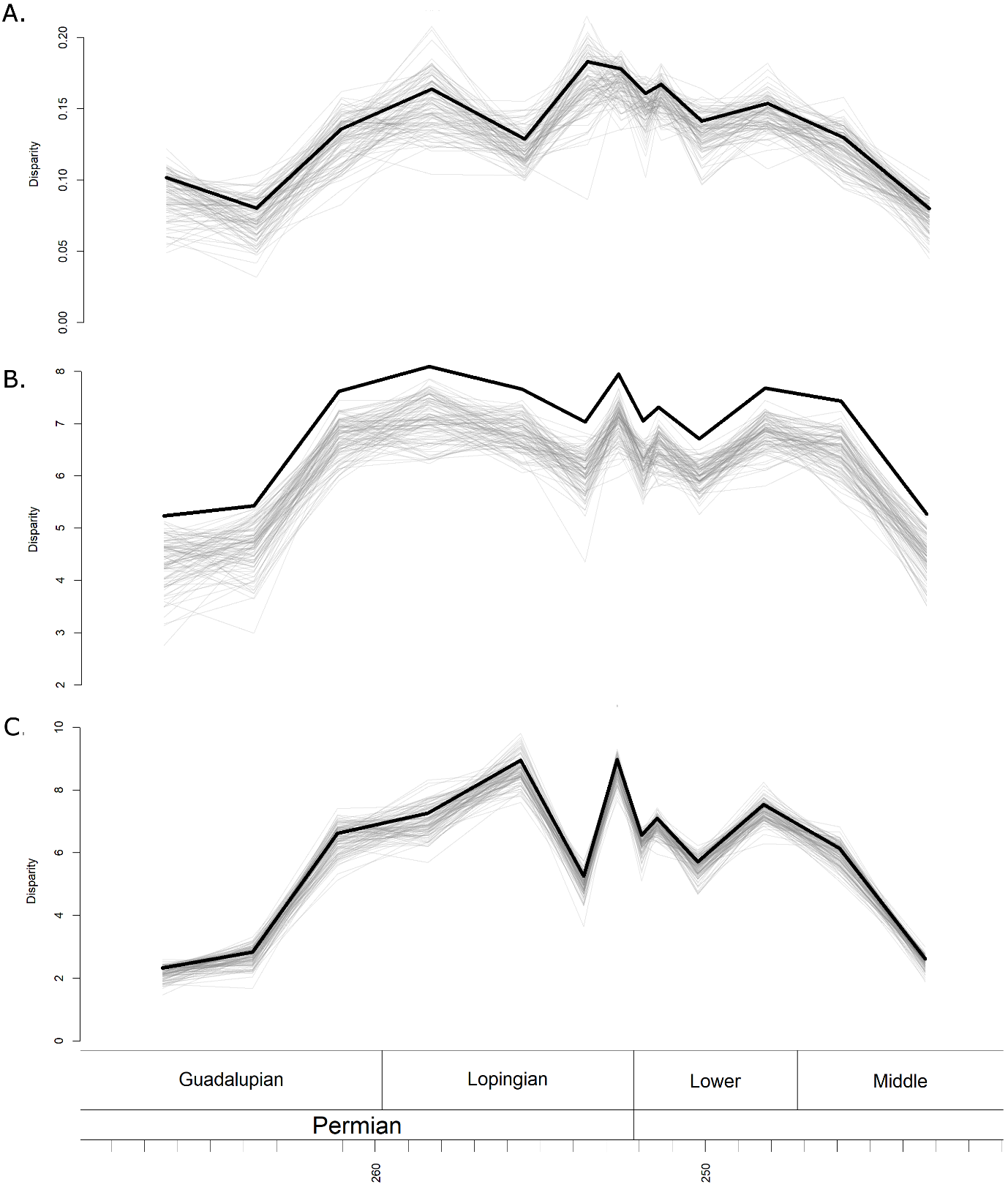


Disparity through time estimated from the maximum clade credibility tree of the analysis where topology is constrained to that of Liu and Abdala [2]. The thick black line represents the raw disparity estimate, the thin translucent grey lines represent disparity estimates from 100 taxonomic bootstrapping replicates. A) Disparity measured using the sum of variance; B) Disparity measured using sum of ranges; C) Disparity measured using mean distance from the centroid.

Supplementary Table S1: The first (FAD) and last (LAD) appearances of the taxa included in this study.

|  | FAD | LAD |
| --- | --- | --- |
| Akidnognathus_parvus | 252.3 | 251.3 |
| Alopecodon | 265 | 259 |
| Annatherapsidus | 252.3 | 251.3 |
| Antecosuchus | 247.2 | 242 |
| Bauria_cynops | 247.2 | 242 |
| Cerdosuchoides | 252.3 | 249.7 |
| Choerosaurus_dejageri | 259 | 254 |
| Chthonosaurus | 252.3 | 251.3 |
| USNM_PAL_412421 | 260.4 | 253.8 |
| Ericiolacerta_parva | 252.3 | 247.2 |
| Euchambersia_mirabilis | 252.3 | 251.3 |
| Glanosuchus_macrops | 265 | 259 |
| Hazhenia | 251.3 | 242 |
| Hofmeyria_atavus | 256.8 | 254.8 |
| Ichibengops | 255 | 255 |
| Ictidochampsa_platyceps | 254.8 | 252.3 |
| Ictidosaurus_angusticeps | 268 | 265 |
| Ictidostoma_hemburyi | 261.5 | 256.8 |
| Ictidosuchoides_longiceps | 254 | 252.3 |
| Ictidosuchops_rubidgei | 254 | 252.3 |
| Ictidosuchus_primaevus | 261.5 | 256.8 |
| Karenites_ornamentatus | 254 | 247.2 |
| Lycideops_longiceps | 254 | 251.3 |
| Lycosuchus_vanderrieti | 265 | 259 |
| Microgomphodon_oligocynus | 247.2 | 242 |
| Microwhaitsia | 260 | 260 |
| Mirotenthes_digitipes | 256.8 | 254.8 |
| Moschorhinus_kitchingi | 252.3 | 251.3 |
| Moschowhaitsia_vjuschkovi | 259 | 252.3 |
| Mupashi | 252.3 | 251.3 |
| Nothogomphodon | 247.2 | 242 |
| Olivierosuchus_parringtoni | 252.3 | 247.2 |
| Ophidostoma | 256.8 | 254.8 |
| Ordosiodon | 247.2 | 242 |
| Pardosuchus | 265 | 259 |
| Perplexisaurus | 260 | 260 |
| Pristerognathus_polyodon | 268 | 259 |
| Promoschorhynchus | 253.8 | 249.7 |
| Regisaurus_jacobi | 252.3 | 247.2 |
| Scaloposaurus_constrictus | 252.3 | 247.2 |
| Scylacosaurus | 265 | 259 |
| Scylacosuchus | 265 | 259 |
| Silphedosuchus | 251.3 | 247.2 |
| Tetracynodon_darti | 252.3 | 251.3 |
| Tetracynodon_tenuis | 254 | 252.3 |
| Theriognathus_microps | 259 | 252.3 |
| Traversodontoides | 247.2 | 242 |
| Urumchia | 251.3 | 247.2 |
| Viatkosuchus_sumini | 260 | 260 |
| Gorynychus_masyutinae | 260 | 260 |
| Shiguaignathus | 259 | 252.3 |

Supplementary References

1. Kammerer, C.F., Masyutin, V. A new therocephalian (*Gorynychus_masyutinae* gen. et sp. nov.) from the Permian Kotelnich locality, Kirov Region, Russia. *PeerJ* **6**:e4933 (2018)
2. Liu, J., Abdala, F. The tetrapod fauna of the upper Permian Naobaogou Formation of China: 1. *Shiguaignathus wangi* gen. et sp. nov., the first akidnognathid therocephalian from China. *PeerJ* **5**:e4150 (2017)
